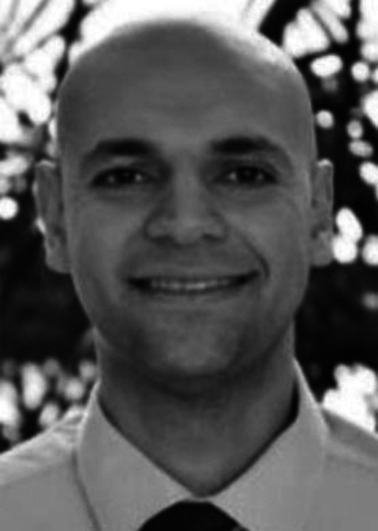# Sustainable Sorbitol Dehydration to Isosorbide using Solid Acid Catalysts: Transition from Batch Reactor to Continuous‐Flow System

**DOI:** 10.1002/cssc.202102525

**Published:** 2022-02-04

**Authors:** Francesco Brandi, Majd Al‐Naji

**Affiliations:** ^1^ Department of Colloid Chemistry Max Planck Institute of Colloids and Interfaces Am Mühlenberg 1 14476 Potsdam Germany

**Keywords:** bio-based building blocks, flow chemistry, heterogeneous catalysis, isosorbide, lignocellulosic biomass

## Abstract

Isosorbide is one of the most interesting cellulosic‐derived molecules with great potential to be implemented in wide range of products that shaping our daily life. This Review describes the recent developments in the production of isosorbide from sorbitol in batch and continuous‐flow systems under hydrothermal conditions using solid acid catalysts. Moreover, the current hurdles and challenges regarding the synthesis of isosorbide from cellulosic biomass in continuous‐flow process using solid acid catalysts are summarized, as well as the scaling‐up of this process into pilot level, which will lead to an established industrial process with high sustainability metrics.

## Graphical abstract

**Isosorbide synthesis in flow**: Isosorbide is a unique bio‐based building block used for a wide series of applications such as pharmaceutics, polymers, and textiles, and development of sustainable process for isosorbide production is greatly required. From this perspective, continuous‐flow systems that operate under hydrothermal conditions represent a potential solution. This Review covers the recent achievements in isosorbide production in continuous‐flow process using heterogeneous catalysts.

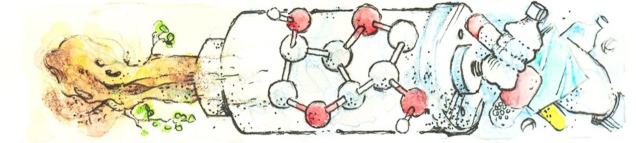

## 1 Introduction

Climate and environmental problems will be the major challenges that humanity will face in the near future. Additionally, these problems will be exacerbated by resources scarcity. Nevertheless, the world demand for energy, materials, plastics, and consumables in general is expected to increase alongside the world population over the next decades. Nowadays, crude oil is our core feedstock for consumable plastics, platform chemicals, and fuels.^[1]^ However, crude oil has several critical drawbacks, such as greenhouses emission if combusted, limited availability, and uneven distribution, leading to economic crises and political conflicts.

In this scenario, the search for sustainable alternatives to crude oil for platform chemicals becomes urgent for chemistry and technology. One of the most promising resources to substitute oil was identified with non‐edible lignocellulosic biomass, which is globally available, renewable, and sustainable.^[2]^ The major component of lignocellulosic biomass is cellulose (35–50 %), and its valorization has a critical importance within the biorefinery processes.^[3]^ Currently, isosorbide is one of cellulose‐derived compounds that gained attention because of its versatility and wide application possibilities. According to the recent United Nation sustainability plans and European Union vision for sustainable and carbon neutral societies by 2030, isosorbide from non‐edible wet cellulosic biomass will be one of the central bio‐based building blocks for the production of fine chemicals and biodegradable polymers.^[4]^

**Scheme 1:**
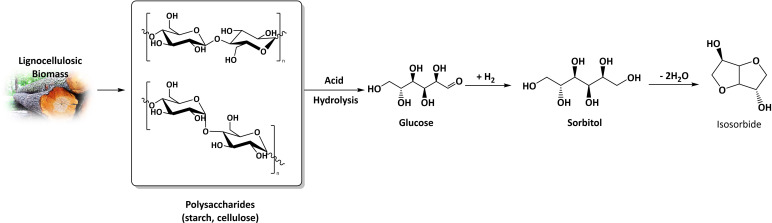
Production of isosorbide starting from lignocellulosic biomass polysaccharides (cellulose or starch), with glucose and sorbitol as intermediates.

Typically, isosorbide is synthetized from sorbitol via a double acid‐catalyzed dehydration reaction.^[5]^ Sorbitol is a lignocellulosic biomass‐derived platform chemical, which is obtained from the sugar fraction of biomass, mostly from starch and cellulose.[^2a^, ^5^, ^6^] To produce sorbitol, starch (or cellulose) are hydrolyzed to glucose, which is hydrogenated toward sorbitol in the following step, generally applying Raney nickel as a catalyst (see Scheme 1).^[7]^

Nonetheless, the transition toward sustainable biorefinery processes should also include the technological process required. In this respect, IUPAC concluded in 2019 that flow chemistry is one of the top ten emerging technologies that will contribute to fulfil the United Nation's Sustainable Development Goals (SDGs), by the year 2030.[^4a^, ^4b^, ^4c^, ^4d^] Flow chemistry processes, with respect to batch counterparts, eventually minimize the risk of handling hazardous and dangerous substances with low cost and increase process efficiency with minimal waste generation, both preventing harm and lowering the environmental impact.^[8]^ Furthermore, flow chemistry allows novel chemical transformations to occur that are not possible in batch.^[9]^

However, flow chemistry in biorefinery is at its infancy and at a laboratory scale.^[9d]^ Nonetheless, efficient industrial biorefining processes are increasingly demanded.^[9d]^ In this regard, our group has reported on the synthesis of a wide range of lignocellulosic biomass‐derived compounds, for example, sorbitol, xylitol, creosol, *p*‐xylene, 2,5‐dimethylfuran, α‐methylene‐γ‐valerolactone, and aromatics from lignin in prototype scale with the potential to reach a pilot scale.[^7b^, ^10^] Additionally, several research groups have reported emerging flow chemistry for biorefinery.[^4a^, ^4b^, ^11^]

The scope of this Review is to provide an updated overview of isosorbide synthesis over heterogeneous catalyst. Moreover, the utilization of continuous‐flow technologies will be discussed as a tool to enable more sustainable isosorbide synthesis. Eventually, the hurdles and future challenges regarding isosorbide synthesis in continuous‐flow systems will be indicated.

## 2 Isosorbide: A Unique Bio‐Based Monomer

Isosorbide, also known as d‐isosorbide, 1,4 : 3,6‐dianhydro‐d‐sorbitol, and 1,4‐dianhydrosorbitol, has white color and crystalline appearance, and high hydrophilicity.^[12]^ It is from the family of diols with a unique two fused furan rings with two secondary hydroxy groups in puckered confirmation (angle of 120°).^[13]^ The C2 hydroxy group extends outside the ring system (*exo*), and the C5 hydroxy group sits inside the “V”shape of the rings (*endo*), with the latter participating in hydrogen bonding with the adjacent THF ring.^[12]^ This hydrogen bond is thought to be at least partially responsible for higher reactivity typically observed for the *endo* hydroxy group.^[12]^ The *endo*‐position hydroxy group is sterically shielded and more acidic than the *exo*‐position hydroxy group. The difference in acidity is explained by the intra‐molecular hydrogen‐bonding between the *endo*‐hydroxy group and the closely located furanic oxygen.^[12]^ This difference between the two hydroxy groups of isosorbide allows selective mono‐functionalization of the diol.[^12^, ^14^]

**Figure 1:**
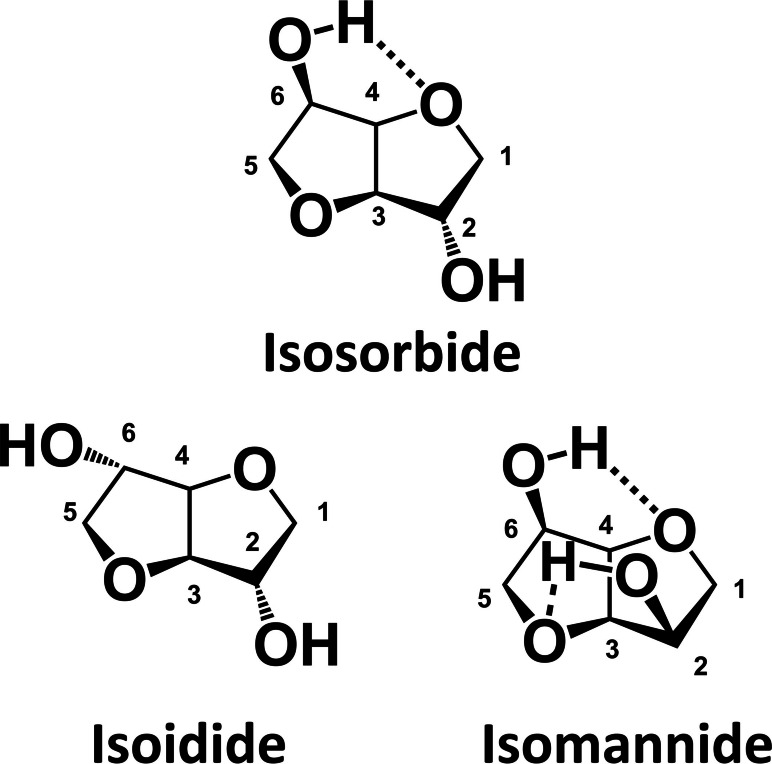
Isosorbide and its stereoisomers: isosorbide, isoidide (1,4 : 3,6‐dianhydro‐l‐iditol), and isomannide (1,4 : 3,6‐dianhydro‐d‐mannitol).

The chemical structure of isosorbide as well as the physical properties are presented in Figure 1 and Table 1. Moreover, isosorbide has two stereoisomers, namely isomannide (1,4 : 3,6‐dianhydro‐d‐mannitol) and isoidide (1,4 : 3,6‐dianhydro‐l‐iditol), as shown in Figure 1.^[15]^ These isomers differ in the spatial configuration of the hydroxy groups in position 2 and 5, being *endo–exo*, *endo–endo*, and *exo–exo* for isosorbide, isomannide, and isoidide, respectively.^[7d]^ Consequently, they present different chemical–physical properties, such as melting point and chemical reactivity.^[7a]^ The hydroxy group in *endo* is chemically shielded due to intramolecular hydrogen bond, and consequently less active, while *exo* hydroxy groups are not sterically hindered and more acidic.[^7a^, ^7c^, ^16^] The simultaneous presence of *endo*‐ and *exo‐* hydroxy groups makes isosorbide suitable for selective functionalization, such as acetylation, tosilation, or alkylation.[^7c^, ^12c^, ^17^] The possibility to undergo selective functionalization makes isosorbide a very promising building block for a new class of bio‐based polymers.

**Table 1:** Physical properties of isosorbide.

Property	Value
chemical formula	C_6_H_10_O_4_
CAS number	652‐67‐5
molecular weight [g mol^−1^]	146.142
physical appearance	white flakes
density at 298 K [g cm^−3^]	1.30
melting point [K]	335–337
boiling point [K]	433
solubility	soluble in H_2_O (>850 g cm^−3^), alcohol, dioxane, ketones

Due to its unique structure, isosorbide can be selectively monotosylated, alkylated, or acetylated, and it presents high rigidity with respect to other flexible diols such as glycols, 1,3‐propanediol, or 1,4‐butanediol.[^12b^, ^12c^, ^17^, ^18^] Furthermore, isosorbide is classified by the US Food and Drug Administration as a non‐toxic substance with a lethal dose (LD_50_) of 24150 mg kg^−1^.^[19]^ All these features make isosorbide one of the most important bio‐based building blocks for the many different fields, that is, polymers, pharmaceuticals, cosmetics, and food industries.[^10b^, ^20^] The global isosorbide market was estimated at USD 413.4 million for 2020 and is projected to grow to USD 703.1 million by 2027.^[21]^

**Figure 2:**
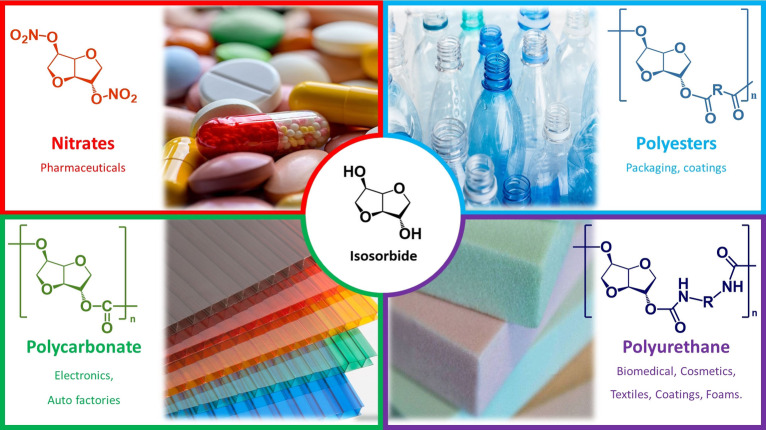
Main isosorbide derivatives with respective applications fields.

Several isosorbide‐based products are already commercialized: polycarbonates such as POLYSORB, DURABIO,^[22]^ and PLANEXT^[23]^ showed superior properties with respect to fossil‐derived polymers, that is, poly(methyl methacrylate) and bisphenol‐A polycarbonate.^[24]^ Moreover, isosorbide copolyesters with terephtalates or furan dicarboxylic acid are a more green and biodegradable alternative to polyethylene terephthalate.^[25]^ Furthermore, the biocompatible and biodegradable isosorbide polyurethanes are suitable for biomedical, cosmetics, and textile applications.[^12c^, ^26^] In addition, isosorbide ethers, such as dimethyl isosorbide, have gained attraction as a green solvent for cosmetics as it is non‐toxic and has low volatility (b.p. 509 K).^[27]^ Beyond materials, isosorbide mono‐ and di‐nitrate are traditionally employed in medicine, especially as vasodilators in cardiologic disease treatments.^[28]^ All these applications are illustrated in Figure 2.

## 3 Current Isosorbide Production Strategy in Batch Reactor

**Scheme 2:**
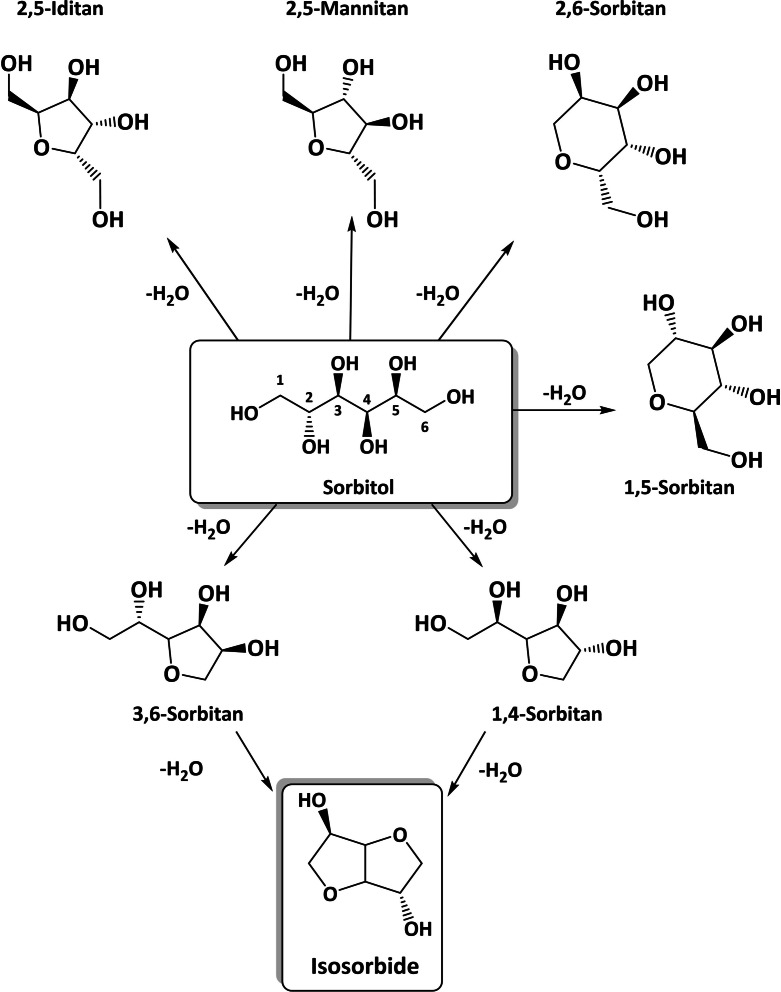
Acid‐catalyzed cyclo‐dehydration pathway from sorbitol to isosorbide via 1,4‐sorbitan and 3,6‐sorbitan.

Isosorbide is produced from the cellulosic fraction of lignocellulosic biomass (mostly starch and cellulose) in a three‐step process, through glucose and sorbitol (see Scheme 1). To form isosorbide, sorbitol has to undergo 1–4‐cyclo‐dehydration followed by 3,6‐cyclodehydration, or vice versa. However, in acidic environment sorbitol can dehydrate toward several mono‐anhydrohexitols (see Scheme 2). Only 3,6‐sorbitan and 1–4‐sorbitan are the possible isosorbide intermediates, while the others mono‐anhydrohexitols are produced as by‐products, namely 2,5‐iditan, 2,5‐mannitan, 2,6‐sorbitan, and 1,5‐sorbitans.^[29]^ Such by‐products can be subjected to degradation and polymerization to humins, which ultimately reduce the mass balance of the reaction.[^7a^, ^7c^] Importantly, several kinetic studies have identified 1,4‐sorbitan as the major byproduct.[^7a^, ^7c^, ^13^, ^29c^, ^30^] Nevertheless, the first sorbitol dehydration selectivity represents the major obstacle toward a quantitative production of isosorbide.^[7c]^ Hwang and co‐workers stated that Brønsted acids are more effective than Lewis to obtain higher isosorbide yield and proposed two different dehydration mechanisms based on different preferential hydroxy protonation in position 1 and 4, respectively.^[29b]^

The double sorbitol cyclo‐dehydration toward isosorbide has been reported in homogeneous phase using both Brønsted acid (*i. e*.,H_2_SO_4_, HCl, and H_3_PO_4_)[^7a^, ^7c^, ^13^, ^29a^] and Lewis acids (*i. e*., AlCl_3_, SnCl_4_, and metal triflates).[^7c^, ^13^] Industrially, isosorbide is produced from sorbitol with 77 % yield using inorganic acid as homogeneous catalyst, mostly using H_2_SO_4_, at 403 K in batch systems.[^7e^, ^13^, ^32^] Nowadays, the major isosorbide producer is Roquette, followed by Archer Daniels Midland, Cargill, Novaphene, Mitsubishi chemical, Sk Chemical, and Par Pharmaceuticals.^[33]^ Homogeneous acidic catalysts present issues in product separation and catalyst recycling; that is, they are non‐sustainable.[^7c^, ^34^] Seeking for more sustainable options, ionic liquids and molten salts have been proposed as reaction media due to their capability of producing isosorbide without any additional cocatalysts.^[7c]^ Accordingly, Li et al. reported 85 % isosorbide yield from sorbitol in molten ZnCl_2_ hydrate salt at 473 K.^[31b]^ Similarly, Brønsted acidic ionic liquids were applied to obtain 73 % of isosorbide yield from sorbitol at 403 K.^[35]^ However, ionic liquids and molten salts have significant issues in terms of products separation and purification, and they can hardly be integrated into the current industrial frame.

For the above‐mentioned reasons, heterogeneous acidic catalysts represent the most promising option to replace homogenous ones, and several different kinds have been proposed for isosorbide production from sorbitol (i. e., zeolites, mixed oxides, acidic carbons, and resins; see Table 2).[^29b^, ^31^, ^36^] Due to the relatively low melting point of sorbitol (368 K), solvent‐free conversion of sorbitol to isosorbide over heterogeneous acidic catalysts has been proposed using a batch system under vacuum with excellent results. Firstly, Fukuoka and co‐workers screened H‐β zeolites with different Si/Al ratio, obtaining 76 % isosorbide at 400 K after 2 h with H‐β(75) zeolite.^[37]^ H‐β(75) zeolite's efficiency derives from its specific textural properties, that is, 3D pore architecture, high specific surface area, Brønsted acid sites, and a pore size (6.6–6.7 Å) slightly larger than the cross section of sorbitol and isosorbide that enables an efficient diffusion of the reactant and product to/from the pores. Similarly, Zhang et al. reported 72 % isosorbide yield at 443 K using sulfonated acidic carbon obtained from waste precursors.^[36d]^ Xiao and co‐workers reported 88 % isosorbide yield using super‐hydrophobic silicon calcined PSSNa composite at 413 K and 2 h of reaction.^[38]^ Kamaruzaman et al. obtained an almost quantitative conversion of sorbitol to isosorbide at 423 K and 4 h of reaction using Amberlyst 36 resin.^[36c]^ Recently, Guo et al. obtained 84 % of isosorbide yield using modified Nb_2_O_5_ at 413 K for 8 h of reaction time.^[39]^ However, the molten‐phase isosorbide synthesis presents some obstacles in view of industrial scale‐up such as the usage of a small amount of catalyst and reactant, the difficulties in product separation, and the discontinuous operational mode. Moreover, the molten‐phase experiment relies on solid and pure sorbitol as reactant and can hardly take place in an integrated process starting from biomass due to the high purification costs.

**Table 2:** Different studies of isosorbide production in batch systems using different solid acid catalysts.

Catalyst	Solvent	*T* [K]	Isosorbide yield [%]	*t* [h]	Ref.
H‐β (75)	melt‐phase, solvent‐free	400	76	2	[37]
sulfonated‐C	melt‐phase, solvent‐free	443	72	2	[36d]
P‐SO_3_‐H	melt‐phase, solvent‐free	413	88	2	[38]
Amberlyst 36	melt‐phase, solvent‐free	423	99	4	[36c]
Nb_2_O_5_	melt‐phase, solvent‐free	413	84	8	[39]
no catalyst	H_2_O	590	57	1	[30a]
H‐β (75)	H_2_O	473	80	10	[40]
BPO_4_	H_2_O	493	72	24	[41]
SiO_2_‐Al_2_O_3_	H_2_O	518	60	24	[42]
H_4_SiW_12_O_40_ and Ru/C	H_2_O	433	17^[a]^	5	[43]
H_4_SiW_12_O_40_ and Ru/C	H_2_O	503	60^[a]^	1	[20a]
Ni‐doped NbOPO_4_	H_2_O	473	47^[a]^	24	[44]

[a] Isosorbide yield obtained starting from cellulose as a starting reactant, whereas the other entries were obtained starting from sorbitol.

As an alternative to melt‐phase reaction, subcritical water has been proposed as a solvent for sorbitol dehydration (see Table 2).[^40^, ^41^, ^42^] Interestingly, Shirai and co‐workers reported isosorbide production in absence of catalyst using water as a solvent, obtaining a maximum of 57 % of isosorbide yield at 590 K after 1 h of reaction time.^[30a]^ Moreover, different solid acid catalyst were investigated in water such as zeolites, SiO_2_−Al_2_O_3_, Nb_2_O_5_, and BP (see Table 2).[^40^, ^41^, ^42^] Similarly to molten‐phase cases, H‐β(75) zeolite showed the best performance also in water phase, with the best isosorbide yield of 80 % at 473 after 10 h of reaction.^[40]^ Commercially, isosorbide is produced mostly starting from edible feedstock, that is, starch.[^7c^, ^7d^, ^45^] However, the usage of edible feedstock is strongly criticized for the economic competition with food and for the soil‐exploitation issues.[^29a^, ^46^] A possible solution is to replace edible starch with inedible lignocellulosic feedstock, mostly cellulose. Palkovits et al. showed firstly the possibility to combine redox catalyst, that is, H_4_SiW_12_O_40_ heteropoly acids, with Ru on carbon as a redox catalyst for hydrogenation reaction, to produce isosorbide from cellulose (isosorbide yield of 17 % at 433 K and 5 MPa of H_2_).^[43]^ With a similar catalyst, Sels and co‐workers reported a 60 % isosorbide yield from cellulose at 503 K and under 5 MPa of H_2_ in batch system combining.^[20a]^ Recently, He et al. reported the direct conversion of cellulose into isosorbide (47 % yield) in water at 473 K over a bifunctional catalyst based on abundant and non‐noble metal, that is, Ni‐doped NbOPO_4_.^[44]^

## 4 Synthesis of Isosorbide in Continuous‐Flow System

Continuous‐flow processes have been increasingly reported as a valid tool to control with high accuracy high temperatures and pressures, as well as to ease the process transition from lab to industrial scale.[^4a^, ^4b^, ^8a^] Additionally, flow chemistry was demonstrated to actively enhance selectivity in reactions involving side products and conformational isomers.^[9a]^ For these reasons, applying flow technology in sorbitol dehydration to isosorbide is particularly interesting.

To date, the synthesis of isosorbide in flow systems has been reported mostly using water steam, that is, vapor‐phase reactions.[^36e^, ^47^] Various solid acids such as sulfated copper oxide,^[47b]^ metal phosphate,^[47a]^ supported tungstophosphoric acid,^[36e]^ and modified tantalum oxide by phosphoric acid was tested in the gas‐phase dehydration of sorbitol to isosorbide (see Table 3).^[47c]^ In vapor‐phase reaction, Cu_2_O(SO_4_) catalyst exhibited the highest isosorbide yield of 68 % at 473 K.^[47b]^ Moreover, a semi‐continuous two‐step process was reported for isosorbide production from cellulose with an overall isosorbide yield of 57 %. This process consists of a first step for cellulose depolymerization–hydrogenation toward sorbitol over Ru/NbOPO_4_ in batch system, followed by a separated second step for steam‐phase continuous‐flow sorbitol‐to‐isosorbide conversion over NbOPO_4_. However, although continuous‐flow vapor‐phase studies reported a good yield of isosorbide, there are several critical drawbacks to a possible scale‐up, such as the additional separation and supply costs of the inert gas as well as the low volatility and thermal stability of sorbitol.^[48]^

**Table 3:** Different studies of isosorbide production from liquid‐ and vapor‐phase sorbitol dehydration in continuous‐flow systems using different solid acid catalysts.

Catalyst	Solvent	*T* [K]	Isosorbide yield [%]	Isosorbide productivity [g_isosorbide_ kg_catalyst_^−1^ h^−1^]	Ref.
H‐β (75)	H_2_O (liquid phase)	503	83	60	[48a]
H‐β (75)	H_2_O (liquid phase)	503	54^[a]^	6.1^[a]^	[48a]
H‐β (38)	MeOH (liquid phase)	473	60	377	[48b]
H‐β (38)	MeOH (liquid phase)	443	28	176	[48b]
Cu_2_O(SO_4_)	H_2_O (vapor phase)	473	68	126	[47b]
PW/SiO_2_	H_2_O (vapor phase)	523	54	216	[36e]
SnPO	H_2_O (vapor phase)	573	47	89	[47a]
H_3_PO_4_ Ta_2_O_5_	H_2_O (vapor phase)	498	47	240	[47c]
H_3_PO_4_ Nb_2_O_5_	H_2_O (vapor phase)	498	63	311	[36e]
NbOPO_4_	H_2_O (vapor phase)	493	50^[a]^	35^[a]^	[49]

[a] Isosorbide yield and its productivity obtained starting from glucose as a starting reactant, whereas the other entries are obtained starting from sorbitol as a starting reactant.

**Figure 3:**
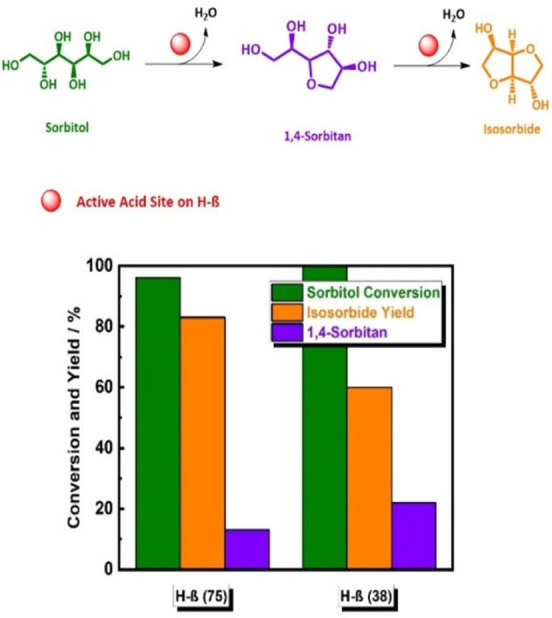
Schematic representation of the continuous‐flow selective liquid‐phase dehydration of sorbitol to isosorbide via 1,4‐sorbitan (top). Conversion of sorbitol and yield of isosorbide and 1,4‐sorbitan using H‐β with Si/Al ratio of 75^17^ in water and H‐β with Si/Al ratio of 38^93^ in methanol (bottom). Reaction conditions using H‐β(75) and H_2_O as a solvent: *c*_sorbitol_=0.05 m, *Q*_solution_=0.3 cm^3^ min^−1^, *T*=503 K, *p*_system_=4.0 MPa, *m*_catalyst_=2.5 g, and space time=3.0 h kg mol^−1^. Reaction conditions using H‐β(38) and methanol as a solvent: *c*_sorbitol_=3.2 wt%, *Q*_solution_=0.2 cm^3^ min^−1^, *T*=473 K, *p*_system_=4.0 MPa, *m*_catalyst_=0.75 g, and contact time=8 min.

Continuous‐flow systems with liquid‐phase solvents are a possible solution to the above‐mentioned hurdles. However, nowadays this option has been still scarcely investigated. Currently, only two studies on continuous‐flow bimolecular dehydration in liquid phase of sorbitol to isosorbide were reported.^[48]^ Our group reported on a continuous‐flow bimolecular selective sorbitol dehydration to isosorbide in liquid water using H‐β(75) with conversion of 94 % and 83 % isosorbide yield at 503 K and 50 h on stream (Figure 3).^[48a]^ Combined with the specific properties of H‐β(75) zeolite, continuous‐flow technology enables fast dehydration of sorbitol to 1,4‐sorbitan, which then is converted to isosorbide. Additionally, flow systems allow continuous and rapid removal of the formed intermediate products from the catalyst surface, which continuously offer free‐acid active sites, which leads to a high yield of isosorbide (83 %; see Figure 3). Furthermore, this process allows rapid access to reaction kinetics, which is greatly needed for process scaling‐up into a pilot level.

One further advantage of liquid phase systems is the possibility to investigate the system kinetics. In our group's work, the rate‐determining step was the second sorbitol dehydration (*E*_a_=125 kJ mol^−1^), while the first was found to be very fast (*E*_a_=55 kJ mol^−1^). In addition, a two‐step isosorbide production from aqueous glucose solution was pioneered (54 % yield) by combining the glucose‐to‐sorbitol hydrogenation step over Ni catalyst supported on nitrogen‐doped carbon with H‐β(75) for sorbitol dehydration to isosorbide.

In parallel to our work, a great study by Hammond and co‐workers was published for synthesis of isosorbide from methanol and water liquid phase of sorbitol over different zeolites, namely H‐β, H‐ZSM 5, H‐Y, H‐FER, and H‐MOR.^[48b]^ This study showed remarkable isosorbide productivity over a long time on stream [176 g_isosorbide_ kg_catalyst_^−1^ h^−1^ using H‐β(38) within 55 h on stream; see Table 3].^[48b]^ Furthermore, this study showed that using methanol as a solvent resulted in high isosorbide and 1,4‐sorbitan yield (60 and 22 %, respectively), as well as enabled this transformation to take part at lower reaction temperature (473 K) compared to water.^[48b]^ Interestingly, diffusion limitation was not observed by performing the experiments at 0.2 cm^3^ min^−1^ with zeolite particle size of 250 μm.^[48b]^

In both studies, the comparison between flow and batch experiments demonstrated a better carbon balance in continuous flow with formation only of 1,4‐sorbitan and isosorbide as products, which indicates the quenching of the competing dehydration reactions (see Scheme 2). This demonstrates that flow chemistry is not only a tool to promote process intensification but also plays a direct role to drive the reaction selectivity. However, to fully understand the exact role of both solvents, that is, methanol and water, and their polarity in continuous‐flow dehydration of sorbitol, additional detailed investigations are still required.

## 5 Conclusions and Future Challenges

The development of innovative technologies for sustainable and efficient isosorbide synthesis is greatly required. Heterogeneously catalyzed reactions have the potential to replace the current homogenously catalyzed process. Noteworthy, the heterogeneous catalysts’ textural properties play a crucial role in the efficient and selective sorbitol dehydration toward isosorbide. H‐β zeolite showed the optimum performance among other solid acid catalysts for the production of isosorbide from sorbitol, due to its 3D pore architecture, Brønsted acid sites, and high specific surface area.

Noteworthy, shifting from batch to continuous‐flow process showed improved productivity of isosorbide. Continuous‐flow technology offers a high tunability of the reaction conditions (temperature, residence time, and pressure), with no diffusion limitation. Furthermore, operating in flow mode resulted in selective dehydration of sorbitol to isosorbide via 1,4‐sorbitan only, whereas the other iditols were suppressed. Besides the more selective isosorbide production, flow systems are inherently safer, easier to scale up, and present more efficient heating. Moreover, the operation of this process in flow chemistry allows connecting multiple tubular reactors, which led to the developing of a more integrated isosorbide production directly from non‐edible cellulosic biomass.

Regardless of the significant advances that have been achieved until now in the field of isosorbide production, some open questions remain without answers and require further detailed investigations. The role of the solvent polarity and its influence on isosorbide yield is still not fully understood and requires further exploration.

The standard solid acid catalyst stability over a long time on stream at elevated temperatures in aqueous media remains the central drawback for the application on a larger scale. Therefore, the development of hydrothermally stable, shaped heterogeneous acid catalysts is necessary.

Microkinetic investigations coupled with operando spectroscopic investigation at a molecular level will assess a deep understanding of this process.

Finally, the transformation of continuous‐flow isosorbide synthesis from a laboratory to a large scale (pilot plant level) with techno‐economic study should be considered to establish the first sustainable industrial continuous‐flow process of isosorbide production in liquid phase.

## Conflict of interest

There are no conflicts to declare.

## Biographical Information

*Francesco Brandi received his Bachelor and Master degrees in Chemistry from the University of Firenze. Currently, he is a Ph.D. candidate at the Max Planck Institute of Colloids and Interfaces under the supervision of Prof. Dr. Markus Antonietti at the Biorefinery and Sustainable Chemistry group. His research focuses on the synthesis of novel catalytic systems for the upgrading of lignocellulosic biomass in flow systems*.

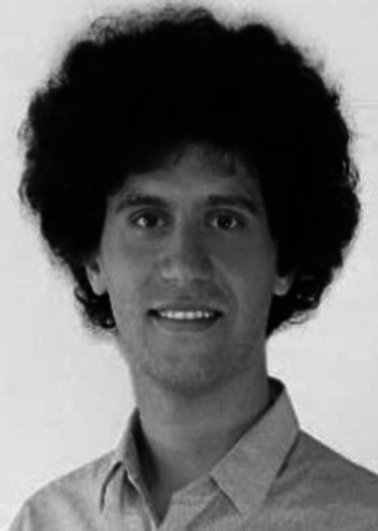

## Biographical Information

*Majd Al‐Naji studied Applied Chemistry at Damascus University (2002–2008). Then, he obtained his Master degree in Structural Chemistry and Spectroscopy from Universität Leipzig (2010–2013). After, he joined the group of Heterogeneous Catalysis in Leipzig under the guidance of Prof. Dr. Roger Gläser (2013–2017). He then worked as postdoctoral researcher at the Sustainable Catalysis and Engineering in KU Leuven with Prof. Dr. Bert F. Sels (2017). Currently, he is leading the “Biorefinery and Sustainable Chemistry” Group at the Max Planck Institute of Colloids and Interfaces*.